# Exploration of Nakivale Refugees' and Stakeholders' Perceptions and Priorities of Male Engagement in Pregnancy, Childbirth, Postpartum, and Family Planning: A Qualitative Study

**DOI:** 10.1155/jp/9050315

**Published:** 2025-10-01

**Authors:** HaEun Lee, Donath Asiimire, Johnson Atwiine, Betrum Namanya, Richard Nsengiyumva, Lynae Darbes, Fred Sheldon Mwesigwa

**Affiliations:** ^1^Department of Systems, Populations, and Leadership, University of Michigan School of Nursing, Ann Arbor, Michigan, USA; ^2^Department of Economics, Statistics, and Tourism Management, Bishop Stuart University, Mbarara, Uganda; ^3^Department of Business, Economics and Governance, Bishop Stuart University, Mbarara, Uganda; ^4^School of Nursing and Midwifery, University of Rwanda, Kigali, Rwanda; ^5^Department of Health Behavior and Biological Sciences, University of Michigan School of Nursing, Ann Arbor, Michigan, USA; ^6^Department of Humanities, Bishop Stuart University, Mbarara, Uganda

**Keywords:** maternal health, men's engagement, men's involvement, refugee health, reproductive health

## Abstract

**Background:**

African refugee communities in Uganda encounter significant barriers to maternal health services, particularly regarding men's involvement in maternal health. This study explored the perspectives of African refugees and stakeholders on men's engagement in couple's maternal health decisions, utilizing an interdependence-based theoretical model as a framework.

**Methods:**

This qualitative study, conducted in Uganda's Nakivale refugee settlement, included 14 in-depth interviews with healthcare providers, community leaders, and religious leaders, along with eight focus group discussions (*n* = 78) with refugee men and women. Participants were purposefully recruited to represent diverse experiences. Data were analyzed through deductive analysis to identify factors influencing men's engagement and couple's behaviors in maternal health, emphasizing predisposing factors that affect motivation and communal coping.

**Results:**

Key individual-level factors influencing engagement included men's financial status, mental health, and peer/community influence. At the couple level, closeness, trust, commitment, communication, and joint household decision-making were crucial for fostering male participation. Couples with a high transformation of motivation viewed maternal health as a shared concern rather than an individual one. Those practicing effective communal coping, discussing and jointly deciding to address maternal health issues, also expressed higher engagement in health-promoting behaviors, such as saving for birth, attending antenatal visits together, utilizing family planning, and sharing household chores.

**Conclusions:**

Maternal health should be reframed as a shared responsibility between partners, not solely women's issue. To effectively engage African refugee couples and improve outcomes, interventions must prioritize men's involvement alongside women-focused efforts, eventually addressing couples together. These initiatives should enhance men's financial literacy, mental health, knowledge, and relationship quality to foster equitable discussions, decisions, and behaviors between refugee couples.

## 1. Background

East Africa hosts approximately 10% of the world's refugees, totaling nearly four million individuals, with women and girls comprising 50.4% of this population [1, 2]. Uganda, one of the largest refugee-hosting countries in Africa, currently accommodates 1.62 million refugees [3, 4]. Refugee and displaced women face significant barriers such as poverty, loss of livelihood, disruption of healthcare services, and the severance of existing social support networks to accessing timely and effective maternal health services [5–7].

While some studies indicate that health outcomes and access to services for refugees may exceed those for host communities due to factors like proximity to health facilities and free services in camp-based settings, refugees still face significant challenges [8–10]. These include cultural differences, communication barriers, discrimination, lack of financial resources, and limited social support [11, 12]. For example, maternal death audits from 43 camps and settlements across eight East African countries highlight that refugee women frequently face high-risk pregnancies and experience substantial delays in seeking, reaching, and receiving care [7]. Additionally, a study on mortality among women in conflict-affected areas across 35 African countries revealed a 21% increase in female mortality, with 10% of these deaths attributable to pregnancy and childbirth [13].

While economic, structural, and sociocultural factors significantly influence refugee women's access to and utilization of maternal health services, one critical factor is the lack of men's engagement [14]. In many African contexts, men are pivotal in decisions related to women's health, including access to reproductive health services, family planning, and financial support for transportation to health facilities [15]. Increased male engagement in maternal health has been linked to improved antenatal care (ANC) attendance, readiness for pregnancy-related complications, better maternal nutrition, higher likelihood of delivery with skilled birth attendants, and enhanced postpartum care attendance [16].

Among African refugee couples, women's access to services such as family planning is often influenced by their husbands [14]. Sociocultural barriers, including the prevailing belief that women are solely responsible for reproductive and child health, further discourage men's involvement [17]. Despite the documented benefits of involving men and lay community members in maternal and child health interventions, there is a lack of research on the role of men in maternal health interventions within conflict settings [13]. This study is aimed at addressing this gap by exploring the perceptions and priorities of African refugees and key stakeholders in Nakivale refugee settlement, Uganda, regarding men's engagement in maternal health.

## 2. Theoretical Framework

Maternal health is often viewed as women's issue, but ideally, pregnancy and childbirth should be considered shared responsibilities between a couple. Both partners should collaboratively discuss, decide, and act to achieve optimal outcomes [18–20]. To explore whether African refugee couples view maternal health as an individual-centered or relationship-centered concern, and to examine the associated predisposing and outcome factors, we used Lewis et al.'s [21] interdependence model of communal coping and behavior change to guide our study. According to this model, couples' *predisposing factors* shape their *transformation of motivation* to address health events as a joint concern rather than as an individual one, leading to a collaborative approach to managing health challenges. Effective *communal coping* involves partners' understanding, discussion, and joint decision-making to effectively address health issues, thereby fostering mutual support and promoting *health-enhancing behaviors*. The model has been widely applied to various health outcomes in sub-Saharan Africa [22, 23]. Given that maternal health is traditionally perceived as women's responsibility, this study is aimed at investigating how African couples can reframe maternal health as a relationship-centered issue and explore how this shift influences communal coping and health-enhancing behaviors.

## 3. Methods

### 3.1. Study Design

We conducted focus group discussions (FGDs) with African refugee men and women of the four most represented nationalities (Congolese, Burundians, Rwandese, and Somali) in Nakivale settlement. We also conducted individual in-depth interviews (IDIs) with key stakeholders, including healthcare providers (midwives, nurses, and physicians), religious leaders (Anglican priests, Catholic priests, and Pentecostal pastors), zone-based community leaders, and the refugee settlement commandant. Group environment of the FGD offers the opportunity to observe and draw on interpersonal dynamics and take advantage of the group dynamics that stimulate discussions [24, 25]. IDIs generate personal narratives from individuals, understanding participants' experiences, beliefs, or opinions on a specific topic [24, 25]. We conducted FGDs among refugee men and women and IDIs with key stakeholders not only to gather enriched and convergent data across the two types of methods but also to mitigate the power imbalance between the different groups of participants [26]. This ensured that refugee men and women can discuss more freely without the community leaders' presence. Data collection utilized semistructured interview guides and a brief demographic survey. All tools were translated in two local languages, Kiswahili and Kinyabwisha, and translated back to English to ensure cross-cultural validation [27]. Kiswahili is commonly used among all four nationalities, and Kinyabwisha is commonly used among Burundians and Rwandese. All data were collected between April and May 2024.

### 3.2. Setting

Nakivale refugee settlement, located in western Uganda, is the oldest and third-largest refugee settlement in the country [4, 28]. With over 178,000 refugees, it is the second most diverse settlement in Uganda, comprising predominantly Congolese (58%), Burundians (21.4%), Rwandese (11.9%), and Somali (8.4%) [29]. The settlement is divided into three zones—Basecamp, Rubondo, and Juru—and hosts seven health centers offering a range of services from basic obstetric care to more complex procedures such as blood transfusions and cesarean sections [30]. The major religious affiliations within the settlement include Catholicism, Anglicanism, and Pentecostalism.

### 3.3. Sampling and Recruitment

All research activities were conducted by our Ugandan research team, consisting of three Ugandan researchers from our collaborating Ugandan academic institution and three research assistants (RAs) of refugee backgrounds. Prior to any research activities, researchers and the RAs attended a two-day in-person workshop where they learned about the study, familiarized themselves with the tools, were trained in the ethical conduct of research, and practiced facilitating IDIs and FGDs.

Participants were purposefully recruited through our research team's established network with community leaders including settlement commanders, healthcare providers, and religious leaders. A settlement commander has semiautonomous authority for managing and supervising the settlement matters [31]. For IDIs, we selected healthcare providers from each health center, two religious leaders from each major faith represented in Nakivale, three zone-based community leaders, and one representative from the settlement commandant's office. Recruitment was facilitated via contact information provided by settlement commanders, healthcare providers, and religious leaders. The research team used a structured script outlining the study's purpose and screening criteria to recruit these key stakeholders. Inclusion criteria for IDI were as follows: (1) above 18 years old and (2) currently working in Nakivale refugee settlement as a religious leader, community leader, settlement commander's office staff, or healthcare provider. Once the participant agreed to participate, the research team agreed on a date and time to meet at their workplace within Nakivale refugee settlement. If the participant disagreed to participate, they were thanked for their time and the team moved on to recruiting the next participant.

FGD participants were also recruited through community leaders and healthcare providers who announced the study in community gatherings such as churches and health centers. Interested individuals shared their contact information with these community leaders and healthcare providers, which was provided to the research team. We aimed for 20 participants (10 from each gender) from each of the four most represented nationalities (Congolese, Burundians, Rwandese, and Somali). FGD inclusion criteria were as follows: (1) country of origin being Democratic Republic of Congo, Burundi, Rwanda, or Somalia; (2) between 18 and 45 years old; (3) married/cohabiting/having been in a committed relationship over the past 6 months; (4) having at least one child under 5 years old; (5) currently residing in Nakivale refugee settlement; and (6) being fluent in either Kiswahili or Kinyabwisha. If the participant agreed to participate, designated date, time, and location were shared. The FGDs were separated out based on gender and nationality.

Despite our original inclusion criteria, information about the FGD spread among other participants who did not meet our inclusion criteria who showed up on the day of FGD. Specifically, few participants were (a) not married, cohabiting, or in a committed relationship over the past 6 months and (b) older than 45 years old. Rather than turning these participants away and causing potential conflicts among refugee members, we deemed that their participation will also provide valuable insights and modified our inclusion criteria to include them. We reported these changes to appropriate ethics boards and ensured that the FGD participants did not exceed the original number planned to be included in the study. The final inclusion criteria were as follows: (1) country of origin being Democratic Republic of Congo, Burundi, Rwanda, or Somalia; (2) older than 18 years old; (3) currently residing in Nakivale refugee settlement; and (4) being fluent in Kiswahili, Kinyabwisha, or English.

### 3.4. Data Collection Procedures

All FGDs and IDIs were conducted by a pair of one Ugandan researcher and one RA. Each IDI lasted approximately 30–45 min, while FGDs ranged from 60 to 90 min. IDIs were conducted at participants' work locations within the settlement, while FGDs were held in communal spaces such as churches and community centers.

The process for both IDIs and FGDs included reading and obtaining written informed consent, followed by a demographic survey. The survey collected data on gender, age, education, occupation, country of birth, nationality, marital status, and number of children. Participants with sufficient literacy completed the survey independently, while those with lower literacy were assisted by facilitators. A semistructured FGD guide was used to facilitate both IDIs and FGDs. It was developed first in English, translated into local languages, and translated back into English to ensure accuracy. The guide included questions exploring participants' perceptions of a healthy man, good husband and father, understanding of maternal health, men's roles in maternal health, and barriers and facilitators for refugee men to be involved in maternal health. The guides also probed existing services and potential interventions to address identified needs. FGDs were conducted separately by gender and nationality to capture cultural nuances and encourage open discussion. The demographic surveys were collected on paper, and all conversations were audio recorded. To thank the participants for their time, we provided 50,000 UGX (approximately $13) for IDI participants. For FGD participants, we provided them with a bar of soap (approximately $3) as well as 50,000 UGX to reimburse their transportation cost.

### 3.5. Data Analysis

At the end of each data collection, Ugandan researchers entered the demographic survey data into an Excel template and uploaded the audio recording to a secure, cloud-based storage. All demographic surveys were then analyzed using Stata (17.0). Descriptive statistics, including counts, means, and percentages, summarized participant demographics.

IDIs conducted in English were transcribed in English. For IDIs and FGDs that were conducted in Kiswahili or Kinyabwisha, transcripts were first produced in the local languages and then translated into English. All transcriptions and translations were cross-checked for accuracy. The final data were imported into Dedoose (Socio-Cultural Research Consultant, LLC) and first independently coded by two researchers to develop an initial codebook (H.L. and D.A.). Then, deductive analysis was conducted by a team of researchers (H.L., D.A., J.A., and B.N.) through iterative team meetings, using the interdependence model of communal coping and behavior change [20] as the analytical framework. We used deductive analysis as it allows the researchers to use existing theory to examine meanings, processes, and narratives of interpersonal and intrapersonal phenomena [32].

## 4. Results

**Figure 1:**
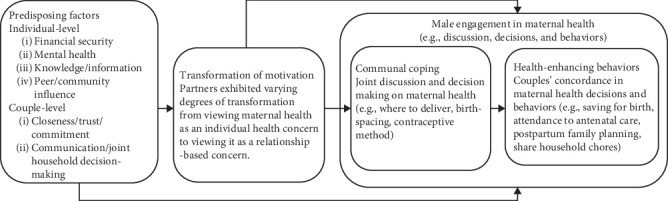
Adapted interdependence model of communal coping and health behavior change for maternal health.

Participant characteristics are presented in Table 1, and the adapted interdependence model [20] based on our study finding is shown in Figure 1. The four themes based on the model are (1) predisposing factors, (2) transformation of motivation, (3) communal coping, and (4) health-enhancing behaviors. Additionally, we identified a theme in (5) desired future intervention characteristics.

### 4.1. Demographic Characteristics

A total of 78 participants were included in the study, 14 IDI participants and 64 FGD participants. The majority of the IDI participants were men (85.7%), between 31 and 45 years old (64.2%), married (85.7%), with tertiary/university or higher-level education (78.6%). Five healthcare providers (35.7%), six religious leaders (42.9%), one representative from the settlement commander's office (7.1%), and two zone-based community leaders (14.3%) participated in the study. Three were Congolese (21.4%), one Burundian (7.1%), four Rwandese (28.6%), and six Ugandan (42.9%). On average, IDI participants have worked and/or lived in Nakivale settlement for 10.3 years (SD, 7.2).

For the FGD participants, gender distribution was almost even, with 31 (51.6%) men and 33 (48.4%) women. Age ranged from 18 to 56 years old. The majority were married (75%) and had primary level or lower levels of education (78.1%). On average, FGD participants desired to have 5.3 children (SD: 2.0) and had 4.3 biological children (SD: 2.5) with the majority of the participants' youngest child being 5 years or younger (70.4%). Six religions were represented, with Anglican (31.2%) and Muslim (26.6%) being the most represented. Nationalities represented included Congolese (25.0%), Burundian (25.0%), Rwandese (23.4%), and Somali (26.6%). Participants lived in Nakivale for 12.8 years (SD, 5.6) on average. Half (51.6%) of them lived in Basecamp, 25% in Juru, and another 23.4% in Rubondo.

### 4.2. Interdependence Model of Health Behavior Change for Maternal Health

Our adapted model (Figure 1) identifies two levels of *predisposing factors* influencing maternal health: individual level and couple level. Individual factors include men's financial stability, mental health, knowledge, and peer/community influence, while couple-level factors involve perceptions of closeness, trust, commitment, communication, and joint decision-making. *Transformation of motivation* was assessed by how participants described pregnancy and family planning—those with high motivation used “we/us/our,” indicating shared responsibilities, while those with low motivation used “she/her” or “he/him,” reflecting separate roles in maternal health. *Communal coping*, couples collaboratively managing health concerns through discussion and joint decision-making, led to *health-enhancing behaviors* such as planning family resources, attending ANC together, adopting family planning, and sharing traditionally gendered household tasks. These findings highlight the interconnected pathways from individual and relational factors to shared health behaviors that improve maternal health outcomes. Unlike the original model of interdependence, we also included *male engagement,* which encompasses both communal coping and health-enhancing behaviors, as maternal health inherently involves women, but men's active engagement enables couples to collaboratively discuss, decide, and act.

### 4.3. Predisposing Factors: Individual Level

#### 4.3.1. Financial Status

Financial status was a key factor in how participants defined a healthy man, a supportive husband, and a caring father. Key stakeholders interviewed in IDIs, as well as both refugee men and women within FGDs, associated a healthy man with his ability to financially provide for the family's needs, such as food, school fees, clothing, and medical bills. The absence of financial stability often led to tension within families and significant stress for men. Many men in Nakivale refugee settlement faced challenges in securing stable income and job, with some having to travel several hours from the settlement for work.

Those who farmed within the settlement noted the insufficient land allotted for sustaining their families and generating income. An IDI participant stated,

These people (refugees) when they are brought here, they are allocated a small piece of land. You have your family, you have your wife, you have your children. They give you a small piece of land like this one, they tell you are supposed to do everything in this area, but it is not enough. (Representative from the settlement commander's office, IDI)

Despite the desire to support their families adequately, including buying birth items ensuring their wives can go to health facilities, financial constraints often limit access to maternal health services. Participants noted significant societal pressure on men to provide as well as internal shame and disrespect from wife and children when they cannot meet these expectations.

… You are broke and the wife, she asks you to give her two thousand (shillings) and you cannot say I don't have since the wife will start saying, “what type of man are you?” And tomorrow the kids would say, “what type of dad are you?” And people will start ignoring you because of that. (Somali man, FGD)

Some women expressed that they would rather have their husbands go to work and make money rather than attending ANC with them. These dynamics underscore the profound impact of financial security on men's health and their roles within their families.

#### 4.3.2. Mental Health

Mental health was a critical predisposing factor that influenced men's perceived ability to function as healthy individuals, as well as good husbands and fathers within refugee communities. Many FGD participants experienced significant mental health challenges, including posttraumatic stress disorder (PTSD), stemming from their experiences of disasters, conflict, and displacement.

… thing that limits a man from being a good husband is because of issue caused by the fact that they are refugees. The first one is war, because war displaced some people… some (of) their children died before them, others lost their parents while physically watching. So, when they reach here in the refugee settlement, anything little disturbs them, they can easily get sick of pressure, and they easily get traumatized mentally. (Rwandese man, FGD)

Furthermore, both men and women in these communities often equated mental health with the ability to maintain peace within the family. A Burundi woman from a FGD shared, *“*a good husband is a calm man, who is on good terms with everyone and stays with others peacefully*”*. Indeed, community members valued men who do not use alcohol or drugs to cope with trauma but maintain tranquility when interacting with their families and others in the community. Such mental and emotional stability among the participants is seen as essential for understanding and adapting to their partner's needs, especially during pregnancy, enhancing their capacity to support their families effectively.

#### 4.3.3. Knowledge and Information

A significant gap in knowledge and information regarding maternal health among African refugee men was noted by study participants. They pointed out that men generally possess less evidence-based knowledge about broad maternal health topics and harbor misconceptions and often lack specific information about their wives' pregnancy and childbirth. Participants stated that men's limited knowledge is due to men being recruited as a secondary audience in maternal health interventions and the current lack of effective interventions primarily targeting men. This exclusion leads many men to feel sidelined in maternal health matters.

… those that teach family planning concentrate on one side. Mostly they talk to women alone, so after taking and teaching them (women)… you, a man, at home later face the consequences of the training acquired by one person without you being involved… (Rwandese man, FGD)

Additionally, IDI participants, including healthcare providers and religious leaders, highlighted that when women withhold specific information about their pregnancy, such as their due date, men are often left uninformed. Moreover, despite prevalent misconceptions about family planning methods within the refugee community, participants noted that men frequently harbor greater misconceptions, which hindered their ability to support their partners effectively.

#### 4.3.4. Peer and Community Influence

The impact of peers and the broader community on men's behavior, particularly in relation to maternal health, was a recurring theme among study participants. Men reported that they often found it easier to express themselves with their peers than with their wives at home. As a religious leader noted, “because some of us, when we're outside here, that's when we talk our feelings. But when we are at home, we tend to hide our issues….”

Further, if a man was seen accompanying his wife to the health facility, community members will tease the man is “bewitched by his wife,” insinuating that the wife is controlling the husband. Alternatively, this meant that men could play a significant role in advocating for and sharing maternal health information within their peer groups.

They can do advocacy and can do information sharing with fellow men. Yeah, because if I'm a man and I've seen the benefits for my wife, then there is no reason why I will not share this same knowledge with the men that are my peers… Men to men, if they share that knowledge amongst themselves and it (maternal health) will improve. (Healthcare provider, IDI)

However, becoming refugees significantly disrupted men's sense of community and social support, impacting how men can support their families. A Somali man described this transition: “While I was in my home country, people support each other… But when we come in a refugee camp, things have changed a lot… You will never see someone help you.” This shift underscores the importance of social networks and community structures in shaping men's involvement in family and health-related matters.

### 4.4. Predisposing Factors: Couple Level

#### 4.4.1. Closeness/Trust/Commitment

Both FGD and IDI participants unanimously agreed that the quality of a couple's relationship significantly influences men's engagement in maternal health and ultimately maternal health. At the couple level, closeness, trust, and commitment were frequently cited as essential components of a healthy and supportive partnership. Expressing affection was described to strengthen family bonds.

A good husband and wife relationship is when you see yourself in your wife… When you love your wife, the children also love you. When I come home, I hug my wife first, and my children laugh. Your wife and children trust you because when you love your wife, you have loved your children too. (Congolese man, FGD)

Participants highlighted the importance of trust and commitment in maintaining a strong marriage. Many noted that a lack of commitment often leads to partners leaving their spouse for someone else, a phenomenon frequently observed in the settlement. Additionally, couples who lack trust were described as prone to constant doubt and suspicion regarding their partner's actions outside the household. A Rwandese woman emphasized the significance of “respecting, loving, and not hiding anything” as essential qualities in a healthy relationship.

#### 4.4.2. Communication/Joint Household Decision-Making

Frequent and ease of communication and shared decision-making were frequently discussed as critical factors of healthy relationships. Participants underscored the importance of couples talking frequently about both big and small things and consulting each other on key family decisions.

… a family is doing well is when a man and woman are united, reason the same way and decide together, that also make a family live better. They decide together on the thing they are to do by name and unite on that. (Congolese woman, FGD)

This collaborative approach to decision-making allows couples to combine their strengths and perspectives, leading to better outcomes for the family. Another Rwandese woman stated, “when you agree at home, you can sit you and your husband and interact, talk about your development your home and cooperate,” emphasizing the sense of unity that comes from making decisions together regarding family's well-being and future development.

### 4.5. Transformation of Motivation

Following the approach by Rogers et al. [23], we analyzed participants' use of “we” versus “I” when discussing maternal health to assess their levels of transformation of motivation. Our findings revealed a spectrum of transformation. Participants who did not exhibit transformation of motivation adhered to traditional gender roles, where maternal health responsibilities, such as attending ANC or seeking guidance from healthcare providers, were seen as “the woman's job.” In these cases, men described their roles as limited to providing financial support and making major decisions, such as choosing the place of delivery or determining family size. Conversely, participants who exhibit transformation of motivation actively engaged in pregnancy and childbirth, perceiving them as shared, relational health responsibilities. These participants emphasized mutual decision-making and support.

Pregnancy emerged as a pivotal period where transformation of motivations occurred, in which traditional roles became more fluid. Men, traditionally viewed as providers, reported taking on household tasks often considered women's responsibilities when their wives were pregnant. For example, a participant shared:

… as a husband, you have to do everything—taking care of the kids, cooking, and ensuring your wife has nutritious food to stay healthy for childbirth. You must save money to provide for her needs and prevent her from carrying heavy things. (Rwandese man, FGD)

These examples highlight that transformation of motivation leads to a redefinition of roles, where pregnancy and childbirth are viewed as shared health concerns.

### 4.6. Communal Coping

In the context of maternal health, participants highlighted in-depth conversations and joint decision-making regarding maternal health. For example, couple's conversations and joint decision-making played a significant role in deciding contraceptive options and family planning. Participants shared how open discussions could help the couples make decisions together about what type of contraception to use, negotiating and compromising to find common ground. However, participants also noted the consequences of a lack of communal coping, breaking families apart due to the consequences of making individual decisions without consulting the significant other. The quotes exemplifying effective and ineffective communal coping are shown in Table 2.

### 4.7. Health-Enhancing Behaviors

When couples effectively discussed and made decisions together, their behaviors related to maternal health improved. These behaviors included the effective planning around family resources for pregnancy and childbirth, joint participation in ANC and checkups, family planning and contraception uptake, and sharing household chores traditionally assigned to women. These examples highlight how joint decision-making positively impacts couples' health-enhancing behaviors, from managing daily household responsibilities to choosing family planning strategies, thereby promoting better maternal and family health outcomes.  Table 3 presents the quotes related to health-enhancing behaviors.

### 4.8. Male Engagement

Unlike prior work on the interdependence model of communal coping and health behavior change, which focuses on outcomes like HIV [22, 23], diabetes [33], and weight loss [34]—where either or both partners may directly experience the health condition—maternal health inherently involves the woman's body, not that of man. Therefore, for couples to effectively engage in communal coping and health-promoting behaviors, men must be first involved. While male engagement is a multifaceted concept with diverse indicators, it often includes communication, decision-making, and behavior [35, 36]. Therefore, we incorporated male engagement in the adapted model to encompass communal coping and health-enhancing behaviors.

### 4.9. Intervention Characteristics

Table 4 highlights the desired characteristics of an intervention targeting men's engagement in maternal and child health among African refugee communities. The participants emphasized the importance of first engaging men in the intervention before involving couples to foster their understanding and responsibility in family health. Cultural and national differences were noted, with suggestions to separate groups by nationality to better address the unique cultural needs of each group. Participants preferred small, intimate group settings to facilitate better understanding and expression of thoughts. Participants also suggested that younger men, including adolescents and young fathers, be included in the intervention and the utilization of community leaders such as religious leaders to increase participant buy-in.

There was also a strong call for integrating financial literacy and income-generating skills to help men become better providers, along with education on household chores, mental health, drug abuse prevention, and intimate partner violence. Many respondents expressed concerns about men's mental health and its impact on family dynamics, while others highlighted the need for addressing intimate partner violence, substance abuse, and teaching men about household responsibilities.

In terms of health content, participants recommended topics like family planning, sexually transmitted infections, and maternal and child health, highlighting the need to be educated on the importance of family planning as well as contraceptive methods, including traditional approaches like the beads method, in which a woman tracks her menstrual cycle with a color-coded string of beads to identify fertile days and nonfertile days. Many participants also saw value in integrating health information into existing community savings groups, which are popular in refugee communities, to incentivize participation and help men better support their families financially.

## 5. Discussion

Our qualitative study examined African refugees' and key stakeholders' perspectives on men's engagement in maternal health, using Lewis et al.'s interdependence model as a framework. In line with this model, our findings highlight several individual- and couple-level predisposing factors that influenced the transformation of motivation and communal coping, which in turn lead to improved health behaviors. These factors are deeply embedded in the socio-political, cultural, and relational dynamics that shape male involvement in maternal and child health.

Financial security emerged as a critical factor, with both men and women defining a healthy man and a supportive husband as someone who can provide for the family's basic needs, such as food, school fees, and medical care. This emphasis on financial provision often limited men's physical engagement in maternal health, such as accompanying their wives to ANC visits [37]. These findings are consistent with studies that highlight the expectation of men being financial providers and that such provision is the main way for men to contribute to maternal health [38]. Additionally, a scoping review identified financial empowerment of men and women to improve men's engagement in maternal health in sub-Saharan Africa [39]. Furthermore, financial insecurity was closely linked to refugee's worse mental health, which further restricted men's involvement in maternal care. Darfur refugees in Chad and internally displaced persons in Uganda both showed that lack of access to basic resources predicted worse mental health outcomes [40, 41]. African refugee men's mental health also significantly influenced their engagement in maternal health. According to Bapolisi et al.'s [42] cross-sectional study examining refugees' perceived psychosocial needs in Nakivale, the most prevalent psychiatric disorder was generalized anxiety disorder (73%), followed by PTSD (67%), major depressive disorder (58%), and substance use disorders. Addressing these economic and psychological barriers is essential to fostering greater male participation in maternal health.

Knowledge and information gaps were also identified as significant barriers to male involvement. Men were often excluded from maternal health interventions, which led to misconceptions and a lack of understanding and participation in maternal health. Furthermore, when women do not share specific information, such as family planning desires and pregnancy due dates, whether due to fear or convenience, men are not able to be effectively engaged in maternal health. Such systematic and personal exclusion perpetuates cultural norms that sideline men in maternal health decisions, limiting their ability to support their families effectively. These findings align with Buser et al. [43] who observed similar gaps in men's access to family planning information. Additionally, indirect learning through peers and other family members rather than direct engagement with healthcare providers contributed to these misconceptions. This highlights the need for direct male engagement in maternal health education through both systematic targeting of men and facilitating conversations among couples. Peer influence plays a substantial role, with many men discouraged from attending ANC by their social networks. A study examining the determinants of male involvement in ANC among refugees in Palabek refugee settlement, Uganda, found more than half (55%) of the participants reported peer influence affecting male partner's involvement in ANC, with 78% of these influences being negative [44]. However, leveraging peer and community influence could help shift these dynamics, leveraging the role social circles can have on men's health decisions [45, 46].

At the couple level, our study emphasizes the importance of friendship, trust, mutual respect, communication, and joint decision-making. This study supports the overall sentiment that couple's relationships significantly influence maternal health [18, 20, 47]. Similar to our study findings, Belus et al.'s [48] study participants in South Africa also identified open communication, communal problem solving, trust, respect, commitment, and connection as important characteristics of healthy relationships. In fact, couples who engage in open communication and joint decision-making in various life events such as children's education and household purchases were better equipped to also discuss and decide on various maternal health–related topics together. This aligns with findings from Uddin et al. [49], who found that couples who shared decision-making and practiced communal problem solving in diverse life decisions such as children's healthcare, household purchases, and visits to family also had higher likelihood to meet maternal health needs [49]. In Uganda's Palabek refugee settlement, frequent discussions about ANC were associated with higher male involvement, underscoring the importance of relationship-centered approaches in health decision-making [44].

The transformation of motivation from an “I” to a “we” perspective was a critical factor in fostering communal coping and shared responsibilities in our study. Similar to Rogers et al. [23], we found that pregnancy was a key time for couples to shift toward a relationship-centered approach to health. Men were more willing to take on traditionally female roles, such as household chores, during their partner's pregnancy, which aligns with findings from Hampanda et al. [38] and Comrie-Thomson et al. [50]. Participants who viewed maternal health as a shared concern (“we/our” pregnancy, childbirth) often spoke about engaging in health-promoting behaviors together, such as attending ANC visits, sharing household responsibilities, and jointly discussing and making decisions about family planning. These findings support existing literature that highlights the importance of shared decision-making and egalitarian power structures in improving maternal and child health outcomes [44, 49].

Future interventions should focus on engaging African refugee men and couples, addressing diverse topics such as financial literacy, income-generating skills, mental health support, and family planning education. Involving community leaders and utilizing existing savings groups were suggested strategies to boost participation and ensure the intervention's success.

This study has several limitations. First, the findings are specific to the Nakivale refugee settlement and may not be generalizable to other contexts. Social desirability bias could have influenced participants' responses, particularly on sensitive topics like family planning and gender roles. Second, while diverse voices were sought, the study may not fully reflect the perspectives of all demographic groups, such as younger men or those less engaged in health services. Third, we modified the inclusion criteria on an ad hoc basis to include participants over the age of 45 who had not been married, cohabiting, or in a committed relationship for the past 6 months. We believe that these adjusted criteria allowed us to capture a broader range of insights. Additionally, the lessons learned from this recruitment process will help refine our recruitment strategy for future studies. Lastly, the reliance on self-reported data limits the ability to assess actual behaviors. Despite these limitations, the study offers critical insights for developing culturally relevant interventions to improve men's engagement in maternal health in refugee settings.

## 6. Conclusion

In conclusion, our study provides critical insights into the perspectives of African refugees and key stakeholders on men's involvement in maternal health, highlighting both the individual and relational factors that influence this engagement. The transformation of motivation from individual to relationship-centered approaches plays a crucial role in fostering communal coping and health-enhancing behaviors. While there are systemic and cultural challenges to male engagement in maternal care, targeted interventions that address these barriers, engage men directly, and promote collaboration between couples offer a promising path forward. Our findings underscore the importance of developing culturally tailored, male-engaged strategies in refugee settings to improve family planning and maternal health outcomes, which can ultimately contribute to healthier, more resilient communities.

## Figures and Tables

**Table 1:** Demographic characteristics of study participants.

	**Total**	**Individual interviews**	**Focus group discussions**
	**N** = 78	**N** = 14	**N** = 64
Gender, *n* (%)			
Men	43 (61.4%)	12 (85.7%)	31 (48.4%)
Women	27 (38.6%)	2 (14.3%)	33 (51.6%)
Age, *n* (%)			
18–20	5 (6.4%)	0 (0.0%)	5 (7.8%)
20–25	10 (12.8%)	1 (7.1%)	9 (14.1%)
26–30	14 (17.9%)	2 (14.3%)	12 (18.8%)
31–35	12 (15.4%)	3 (21.4%)	9 (14.1%)
36–40	13 (16.7%)	3 (21.4%)	10 (15.6%)
41–45	15 (19.2%)	3 (21.4%)	12 (18.8%)
> 45	9 (11.5%)	2 (14.3%)	7 (10.9%)
Marital status, *n* (%)			
Married	60 (76.9%)	12 (85.7%)	48 (75.0%)
Divorced/separated/widowed	7 (9.0%)	1 (7.1%)	6 (9.4%)
Single/never married	11 (14.1%)	1 (7.1%)	10 (15.6%)
Education level, *n* (%)			
Never went to school	16 (20.5%)	0 (0.0%)	16 (25.0%)
Primary	34 (43.6%)	0 (0.0%)	34 (53.1%)
Secondary	12 (15.4%)	3 (21.4%)	9 (14.1%)
Tertiary/university or higher	16 (22.9%)	11 (78.6%)	5 (7.8%)
Job, *n* (%)			
Farming	34 (43.6%)	0 (0.0%)	34 (53.1%)
Raising animals	1 (1.3%)	0 (0.0%)	1 (1.6%)
Cleaning/washing clothes/digging	15 (19.2%)	0 (0.0%)	15 (23.4%)
Teacher	1 (1.3%)	0 (0.0%)	1 (1.6%)
Healthcare worker	5 (6.4%)	5 (35.7%)	0 (0.0%)
Business	8 (10.3%)	0 (0.0%)	8 (12.3%)
Bicycle/motorcycle taxi	1 (1.3%)	0 (0.0%)	1 (1.6%)
Pastor/priest/religious leader	7 (9.0%)	6 (42.9%)	1 (1.6%)
Settlement commander	1 (1.3%)	1 (7.1%)	0 (0.0%)
Zone-based local leader	2 (2.6%)	2 (14.3%)	0 (0.0%)
Other	3 (3.8%)	0 (0.0%)	3 (4.7%)
Desired number of children, *n* (SD)	5.2 (2.0)	4.6 (2.4)	5.3 (1.8)
Number of biological children	4.1 (2.4)	3.6 (2.5)	4.3 (2.3)
Number of total children	4.5 (2.5)	4.4 (2.7)	4.6 (2.5)
Age of the youngest child			
6 months or younger	7 (9.0%)	1 (7.1%)	6 (9.4%)
6 months–1 year	11 (14.1%)	0 (0.0%)	11 (17.2%)
1 year–5 years	38 (48.7%)	10 (71.4%)	28 (43.8%)
Older than 5 years	14 (17.9%)	1 (7.1%)	13 (20.3%)
Not applicable^a^/missing	8 (10.3%)	2 (14.3%)	6 (9.4%)
Religion, *n* (%)			
Anglican	26 (33.3%)	6 (42.9%)	20 (31.2%)
Pentecostal	8 (10.3%)	3 (21.4%)	5 (7.8%)
Catholic	12 (15.4%)	3 (21.4%)	9 (14.1%)
Muslim	18 (23.1%)	1 (7.1%)	17 (26.6%)
Seventh-day Adventist	6 (7.7%)	1 (7.1%)	5 (7.8%)
Baptist	8 (10.3%)	0 (0.0%)	8 (12.5%)
Country of origin, *n* (%)			
Democratic Republic of Congo	19 (24.4%)	3 (21.4%)	16 (25.0%)
Burundi	17 (21.8%)	1 (7.1%)	16 (25.0%)
Rwanda	19 (24.4%)	4 (28.6%)	15 (23.4%)
Somalia	17 (21.8%)	0 (0.0%)	17 (26.6%)
Uganda	6 (7.7%)	6 (42.9%)	0 (0.0%)
Years lived/worked in Nakivale refugee settlement, *n* (SD)	12.3 (6.0)	10.3 (7.2)	12.8 (5.6)
Zone living in Nakivale, *n* (%)			
Basecamp	39 (50.0%)	6 (42.9%)	33 (51.6%)
Juru	20 (25.6%)	4 (28.6%)	16 (25.0%)
Rubondo	18 (23.1%)	3 (21.4%)	15 (23.4%)
Missing	1 (1.3%)	1 (7.1%)	0 (0.0%)

^a^Not applicable if a participant does not have any children.

**Table 2:** Quotes related to effective and ineffective communal coping.

**Communal coping**	
Effective	“… when a man and a woman talk and decide on family planning methods… they sit down and decide that since (a specific type of) family planning has failed, let us use condoms. But between them, one may not buy the idea… one says, ‘I do not want it (condom).' Then the man tries and leaves you alone and says, ‘woman, let me leave you alone, do not cause me problems of maybe making you pregnant again… So it helps him take care very well based on the decisions we both made.'” (Congolese woman, FGD)
Ineffective	“I know one family… the husband told the wife that he did not want a third child… the wife conceived, and the daddy immediately left the family… they are there struggling with life because they produced without planning.” (Zone-based community leader, IDI)

**Table 3:** Quotes related to health-enhancing behaviors.

**Health-enhancing behaviors**	
Use family resources for maternal health	“…. Immediately after conception, you get to know how to take care of the pregnancy up to the time of birth. You look out for the baby's clothes at birth, get to know that it shall need a baby shawl to carry the baby…” (Congolese man, FGD)
Joint attendance in ANC and checkups	“The first thing is supporting her and accompanying getting to the doctor and do all the tests related to pregnancy.” (Burundi man, FGD)
Uptake of contraceptive methods	“To me, the way we do it, although most times I do not see it here in hospitals, is by using the beads method. It was so helpful without taking any tablet or injection… After your periods, you would know the days to spend before meeting up with a man based on the way you agreed and understand each other.” (Congolese woman, FGD)
Sharing household chores	“when he knows that the wife is pregnant, he does not let her do heavy work and gives her food that prevent diseases including greens and are on the side of the wife. It makes the baby inside to feel well.” (Burundi woman, FGD)

**Table 4:** Quotes related to desired intervention characteristics.

**Desired intervention characteristics**	
Target audience/group characteristics	
Engage men first	“Actually, now days, men are saying that they are left behind. All programs that come are empowerment for women, not empowerment for men. So like if we get like partners to come on ground and they teach men what they are supposed to do how they can live well with their family members.” (Zone-based community leader, IDI)
Engage couples later	“… From the start, I still recommend you have men purely (only), then in subsequent meetings then we can bring women. But you have first dealt with men, but later we can involve women because mainly with family planning, man will just make the women go.” (Healthcare worker, IDI) “They should be together, should be together, because when they are together, because when they express their views and concern, when they are with their women applying those knowledges, it helps. It helps the wife, the wife wants to be supported. Because, you can teach men separately from their wives, and when they reach home, a man behave like they learned nothing, knowing that the woman was not there and she does not know what he has been taught so let him continue in his way. But when they are together, yeah there is no there is no decision a man can make apart from the woman.” (Religious leader, IDI)
Separate groups based on nationality	“What works for this group, may not work for this other group due to the cultural difference. Yes, for example, a Congolese man may not understand the perspective and the way around other men will understand it. Yeah, so if you bring all of them together you may not have achieved anything, or you may achieve very minimal. So, it is better they are grouped them either by nationality or by age group so that you give them information that targets their specific categories.” (Healthcare worker, IDI)
Intimate group setting	“I feel they should start with small groups like 15 to 30 people so they start when they are few so that they are reached out to.” (Religious leader, IDI) “Yeah, information sharing is better with smaller groups, because when we share information to masses, we cannot check understanding. It becomes hard… Even expression of their feelings or their thoughts about particular program will be hard… so small groups of maybe 15 to 20 and sessions lasting not more than an hour.” (Healthcare worker, IDI)
Pairing with savings groups	“… here are many organizations around that go giving support in groups, you find them investing money in groups and that money helps them. But mostly those groups are over occupied by women. As also as men, we would wish like you asked that if you are to make those groups, should they be of like 10 or 30 people… You know men we love thinking a lot, we go around here and there and look for what to do as you also help us and put money in our saving groups if possible so that the money helps us get more money.” (Congolese man, FGD) “… the trainings about how one can save for emergencies. They say that maybe in this village we want like 20 men or 30 to make a saving group, at least every week save one thousand (Ugandan shillings) each,…” (Congolese woman, FGD)
Involvement of younger adults	“… Here we have a seventeen-year-old men with babies. You find a nineteen-year-old carrying a baby of a twenty-year-old… specifically (need an intervention) for men these other ones, outside the bracket (of reproductive age 18-45), of adolescents.” (Healthcare worker, IDI)
Involvement of community leaders	“I was looking at the community part, like religious leaders. They can play a they very big role in assuring that there is harmony in our homes and that individual level. Also had local leaders. So the local leaders are also very very important. There should also be healthcare providers, us they have always been there for these families because of any challenge when it comes to maternal child health. And also looking at the health of men.” (Healthcare worker, IDI)
Content	
Financial literacy	“Because many families have been separating am telling you due to silly things, so men are totally ill mentally. So that program can awake the minds of men even it can also rebuild like those men who have lost their hope and also I see this financial literacy it's a key topic that can also help men to build themselves and also think for the family. In this financial literacy. I think there is an organization comes and forms like these saving groups after financial literacy, from saving groups it will help men those who are going like to borrow money from banks, loans from other micro finance institutions they can resort to groups because groups have low interest rates.” (Zone-based community leaders, IDI)
Income-generating skills	“The second thing is skills, like right now we are here being encouraged to go on as the first thing. So for us now, girls come and are trained some skills yet most boys are not considered for the trainings. They say that it's a must to learn tailoring and for us boys, we are discriminated on that for they don train us.” (Congolese man, FGD) “Like training us vocational skills and support us in those so that we can rise up having something we do for us to increase and develop.” (Rwandese man, FGD)
Household chores	“they [men] need to learn how the home operates such that children can also learn from them.” (Rwandese woman, FGD)
Mental health	“… men are totally ill mentally. So that program can awake the minds of men even it can also rebuild like those men who have lost their hope” (zone-based community leader, IDI)
Drug/substance abuse	“Another thing I can add on is that you can be a man and engage in drinking alcohol…and another thing there are drugs. They take like marijuana and mairungi (plant that can cause cognitive stimulation and euphoria), so some men you have joined the taking of such drugs become unhealthy because their lives have been damaged by those things.” (Congolese man, FGD)
Intimate partner violence	“There is a time he drinks alcohol; he comes home the wife and children they do not get peace he reaches there beating them up.” (Burundi woman, FGD) “They greet (when coming back home), do not beat wives after learning.” (Rwandese woman, FGD)
Couple's conflict resolution	“A wife and husband relationship can be affected when they two fight in front of the children because children also get a problem after. So, if a family conflicts all the time, children also decide to leave that home and run away… A family that is not united most especially a man and a woman, children get disorganized and also start conflicting due to lack of moral upbringing.” (Congolese man, FGD) ”… One topic to be add in the program conflict resolution in the family and others who have been separated and bringing many people to solve their issue.” (Somali man, FGD)
Sexually transmitted diseases	“… Maternal child also involved the component of addressing sexual reproductive, for example, STI screening of giving them condoms, testing for HIV, those topics should be discussed on there.” (Healthcare provider, IDI)
Family planning	“It's also a family planning method… So I tell you, A contains this this, B contains this this, the side effects can be this this, but there are options if maybe you have regular blooding we can first do this, but if you feel its inconveniencing you, you can stop. There is also criteria for stopping. And also there is a criteria as you say, like exclusion criteria for a woman who has diabetes, who has (high blood) pressure, should not be given maybe pills, and implants this like that. So I give you the options from what I have told you, you be like let me try an IUD because maybe for it does not have hormones, just blocks the tubes such that.” (Healthcare worker, IDI) “To me the way we can do it, although most times I do not see it here in hospitals, but there is a way we used to study on beads and it was so helpful without taking any tablet or injection. So, if they are black beads you would count starting from today, tomorrow and the other day until you finish the black ones, when you would reach on the red bead you would get to know that tomorrow you might see your periods and prepare on how to conduct yourself. So after your periods you would know the days to spend before meeting up with a man based on the way you agreed and understand each other. So between the seven days and fourteen days, after those days you now continue with your plans with your man as usual but I found that that method of beads is not there.” (Congolese woman, FGD)
Maternal and child health	“We are engaging the woman during immunization we are engaging, her during antennal, we are engaging women during all those programs. But we understand that there is a very small percentage at which we are engaging men, so if there is widening with this scope this program is designed to include a comprehensive package of how a man participates in maternal child health, like information sharing sessions and everything to do with that.” (Healthcare worker, IDI)

## Data Availability

The data that support the finding of this study are available from the corresponding author upon reasonable request.
